# Milieu matters: Evidence that ongoing lifestyle activities influence health behaviors

**DOI:** 10.1371/journal.pone.0179699

**Published:** 2017-06-29

**Authors:** Rob Lowe, Paul Norman, Paschal Sheeran

**Affiliations:** 1 Swansea University, Swansea, United Kingdom; 2 University of Sheffield, Sheffield, United Kingdom; 3 University of North Carolina at Chapel Hill, Chapel Hill, North Carolina, United States of America; IRCCS Istituto Auxologico Italiano, ITALY

## Abstract

Health behaviors occur within a milieu of lifestyle activities that could conflict with health actions. We examined whether cognitions about, and performance of, other lifestyle activities augment the prediction of health behaviors, and whether these lifestyle factors are especially influential among individuals with low health behavior engagement. Participants (*N* = 211) completed measures of past behavior and cognitions relating to five health behaviors (e.g., smoking, getting drunk) and 23 lifestyle activities (e.g., reading, socializing), as well as personality variables. All behaviors were measured again at two weeks. Data were analyzed using neural network and cluster analyses. The neural network accurately predicted health behaviors at follow-up (*R*^*2*^ = .71). As hypothesized, lifestyle cognitions and activities independently predicted health behaviors over and above behavior-specific cognitions and previous behavior. Additionally, lifestyle activities and poor self-regulatory capability were more influential among people exhibiting unhealthy behaviors. Considering ongoing lifestyle activities can enhance prediction and understanding of health behaviors and offer new targets for health behavior interventions.

## Introduction

Research into predictors of health behaviors tends to focus exclusively on cognitions concerning the behavior of interest, and eschews questions about other ongoing lifestyle activities [1]. Researchers interested in exercise ask questions about exercise behavior, those interested in smoking ask questions about smoking, and researchers concerned with alcohol consumption enquire about drinking behavior [2]. The rationale for focusing on behavior-specific cognitions comes from models such the theory of planned behavior [3–6] which assume that variables not specified by the model, such as other ongoing activities, only affect behavior via their influence on behavior-specific cognitions. However, daily life demands that people manage multiple intentions and behaviors [7–10]; performance of any particular health behavior occurs within an interdependent set of activities undertaken during the same time period. The present study tests whether cognitions about, and performance of, other ongoing lifestyle activities enhances the prediction of focal health behaviors beyond the influence of behavior-specific intentions, attitudes, norms, and perceived behavioral control. We also examine whether other ongoing lifestyle activities are especially influential for people who do *not* engage in health behaviors.

### Predicting health behaviors

Several theories including the theory of planned behavior, protection motivation theory, and the health action process approach have been developed to specify the factors that predict health behaviors (see [2] for a review). A recent analysis of 10 such health behavior theories indicated that the models converged on *intentions*, *attitudes*, *norms*, and *perceived behavioral control /self-efficacy* as key predictors [11]. Intentions summarize people’s motivation to act and are construed as the most immediate and important predictor of health behaviors. Intentions, in turn, are determined by the person’s overall evaluation of the consequences of performing the behavior (attitude), perceived social pressure to act (subjective norm), and perceptions of the act’s ease or difficulty (perceived behavioral control, PBC). Meta-analyses indicate that these four predictors account for 14% to 24% of the variance in health behaviors [6, 12]. However, a large proportion of behavior variance is unexplained [13] suggesting a role for other predictor variables. Several studies have tested whether additional cognitive constructs such as descriptive norms and self-identity improve behavioral prediction (reviews by [14–16]). However, these additional constructs also concern the focal behavior. The possible significance of other ongoing activities for predicting health behaviors has largely been ignored.

### The role of other ongoing activities

There have been calls for a deeper analysis of how health behaviors fit into wider lifestyle choices and for an enlargement of health behavior theories to accommodate facilitating and conflicting goals (e.g., [17, 18]). Daily routines require managing multiple goals and behaviors during scheduled time periods [19]. Health behaviors are part of this milieu of ongoing activities. Enactment of health behaviors could be influenced by cognitions about, and performance of, these other lifestyle activities in two main ways. First, ongoing activities could facilitate performance of health behaviors. Facilitation can occur (a) because the behaviors overlap, i.e., performing the ongoing activity involves performing the health behavior; for instance, taking a long walk in the countryside entails exercise behavior, or (b) because the behaviors are related instrumentally; for example, reading for pleasure or watching a video could constitute strategies that enable someone to avoid getting drunk [20, 21]. Second, ongoing activities could conflict with, and so inhibit, focal behaviors [22]. Resource limitations may require prioritizing one behavior over another, or other ongoing activities may be incompatible with a focal health behavior. For example, it is not possible to read for pleasure and engage in exercise at the same time [19]. Additionally, ongoing activities could deplete self-control resources and so compromise the effective pursuit of health goals. For example, forcing oneself to study over an extended period could make it hard to resist a tempting snack [23]. In sum, factors such as behavioral overlap, instrumental relations, behavior compatibility, and material or self-control resources may determine whether ongoing lifestyle activities enable or inhibit enactment of health behaviors.

To date, only a small body of research, predominantly focusing on physical activity, has examined how health behaviors fit into wider lifestyle choices. For instance, conflicting behaviors predicted less physical activity over and above the effects of physical activity intentions and perceived behavioral control [24]. Similarly, intentions to watch TV significantly predicted less physical activity two weeks later, even after accounting for behavior-specific cognitions [25]. However, other studies have failed to observe significant relationships between perceived goal conflict and physical activity [26, 27]. It appears that the case is not yet made that ongoing lifestyle activities have an important influence on health behavior performance.

### The present research

We extend previous studies concerning the influence of other ongoing activities on focal health behaviors in three key ways. First, whereas previous studies assessed a single health behavior (physical activity), the present study considers a suite of health behaviors. We focus simultaneously on smoking, excessive alcohol consumption, dietary behaviors, and exercise because these behaviors have substantial effects on mortality and morbidity (e.g., [28–30]). Assessing several focal behaviors simultaneously also reflects the reality that people undertake multiple health behaviors as well as multiple other lifestyle activities. Second, the present study examined a large and unbiased set of ongoing lifestyle activities. Twenty-three other behaviors that participants could undertake during the same follow-up period as the focal health behaviors were specified. These other activities were unbiased in that (a) no attempt was made to select behaviors that were likely to conflict with or facilitate performance of the focal behaviors, and (b) participants were not sensitized to the aims of the research by assessing *perceptions* of goal conflict or facilitation. Third, the present study examined both the overall impact of ongoing lifestyle activities on focal behavior performance and individual differences in how much influence was exerted by those other activities.

Our individual difference analyses were inspired by counteractive control theory (e.g., [31]) which proposes that people who successfully pursue focal behaviors are likely to have developed efficient mechanisms for managing conflicting activities. Conversely, people who engage less in focal behaviors are reliant on conscious, effortful shielding of those behaviors from alternative activities, and so are more likely to be tempted by distractions. Fishbach et al. [31] showed that for participants good at controlling their weight, priming competing goals such as tempting chocolates automatically activated weight control goals. For participants who were poor at self-regulating their weight, on the other hand, priming engendered no activation of the weight control goal. Thus, we predicted that there would be a negative relationship between undertaking other lifestyle activities and focal health behaviors, but only for participants who infrequently perform health behaviors. Consistent with Fishbach et al.’s analysis [31], we also predicted that people who engaged less in health behaviors would be characterized by low scores on key personality variables relevant to goal management [32, 33]: generalized self-efficacy, action control, self-esteem, and locus of control. Self-efficacy encompasses a person’s belief about their ability to overcome obstacles to a desired goal, and determines effort exerted in achieving the goal [34]. Action control refers to people’s ability to regulate interfering emotions when striving to achieve a desired goal [35]. Self-esteem refers to people’s attitude toward themselves and is shaped by life experiences, including accomplishments and failures [36]. Locus of control refers to the degree to which people believe they are instrumental in causing life events, and the extent to which these beliefs are reinforced when outcomes are or are not consistent with such beliefs [37].

In sum, the present study deployed a series of innovations to test whether other ongoing activities influence performance of focal health behaviors. Two hypotheses were tested: (1) cognitions about, and performance of, other ongoing activities would predict health behaviors over and above the influence of behavior-specific cognitions, and (2) participants with low engagement in health behaviors would (a) be especially liable to influence by other ongoing activities, and (b) exhibit poor self-regulatory capability according to standard personality measures.

## Method

The originating study was approved by the Psychology Research Ethics Committee at the University of Sheffield (UK) where data collection was managed and followed the ethics guidelines specified by the British Psychological Society. Participants were informed that taking part in the research was voluntary and that completing the questionnaires would be taken to indicate consent. Participants were instructed not to sign the questionnaire. An anonymous code was used to match responses from both time-points.

### Participants and procedure

A longitudinal questionnaire survey of 28 behaviors was conducted among a convenience sample of 211 Psychology undergraduates (68.7% women, *M*-age = 20.26, *SD* = 2.92). At baseline, participants completed a questionnaire that measured cognitions about, and frequency of performance of, five health behaviors and 23 other lifestyle behaviors, as well as several personality factors. Two weeks later, participants completed a follow-up questionnaire concerning their frequency of performing the 28 behaviors (see Table 1 for overview of study variables).

**Table 1:** Variables measured at each time-point.

Time-point/Construct		Variables measured	Sample items
*Baseline*			
Health behaviors (past behavior)	Five specific health behaviors		“How many times have you [performed the behavior] over the last two weeks?”
Health behavior cognitions; Ongoing lifestyle cognitions	Attitude		“[Performing the behavior] over the next two weeks would be…” (unpleasant-pleasant)”
	Subjective norm		“Most people who are important to me think I should [perform the behavior] over the next two weeks …” (unlikely-likely)
	Perceived behavioral control		“For me to [perform the behavior] over the next two weeks would be …” (very difficult-very easy)
	Intention		“I intend to [perform the behavior] in the next two weeks…” (definitely don't-definitely do)
Personality factors	Locus of control scale		“When I make plans, I am almost certain that I can make them work”
	Action control scale (ACS)—Preoocupation		“When I have lost something valuable and can’t find it anywhere: I have a hard time concentrating on anything else”
	Action control scale (ACS)—Hesitation		“When I know I must finish something soon: I have to push myself to get started”
	Rosenberg self-esteem scale		“On the whole I am satisfied with myself”
	Generalized self-efficacy scale		“It is easy for me to stick to my aims and accomplish my goals”
*Follow-up*			
Health behaviors	Five specific health behaviors		“How many times have you [performed behavior] over the last two weeks?”
Ongoing lifestyle activities	Twenty-three specific behaviors		“How many times have you [performed behavior] over the last two weeks?”

#### Behaviors

We identified behaviors from a pilot test of lifestyle activities among 31 UK undergraduates. The final set of behaviors was selected on the basis that approximately one-half of the participants engaged in the behaviors at the specified frequency in the previous two weeks. Baseline and follow-up behaviors were assessed with items devised specifically for the study. Participants were presented with the stem, “How many times have you [performed the behavior] over the last two weeks?” (with minor variations), and entered the number in a space provided. The study examined five *health-related behaviors*: engaging in at least 20 minutes of moderate/vigorous exercise, smoking cigarettes, getting drunk, number of days had eaten at least one piece of fruit, and eating fast food. Twenty-three *other lifestyle activities* were assessed: buying a magazine, buying a newspaper, reading for pleasure, taking vitamin pills, visiting a friend, going out for a meal, attending all lectures, getting at least 7 hours sleep, buying clothes, not lying-in past 9.00am, going to the cinema, going for a walk, engaging in independent study, writing a letter, recycling bottles, visiting the countryside, going to the library, avoiding eating meat, renting a video, going shopping, visiting parents, going clubbing, and tidying room. Two additional behaviors were assessed (going to the sports center, going to the pub) but were not included in the analyses because they overlap too greatly with the target health behaviors of exercise and avoiding getting drunk, respectively.

#### Cognition measures

Cognitions were measured on 7-point semantic differential scales. Item wording followed the standard format for measuring each construct (e.g. [38]). *Intention* was assessed by two items (e.g., “I intend to [perform the behavior] in the next two weeks…”; definitely don't-definitely do). *Attitude* was measured by three items “[Performing the behavior] over the next two weeks would be…” (bad-good, unenjoyable-enjoyable, unpleasant-pleasant)”. *Subjective norm* was measured by two items (e.g., “Most people who are important to me think I should [perform the behavior] over the next two weeks…”; unlikely-likely). *Perceived behavioral control* (PBC) was measured by four items (e.g., “For me to [perform the behavior] over the next two weeks would be …”; very difficult-very easy). The internal reliability of TPB variables for the 28 behaviors was high (median alphas = .97, .82, .92, and .83, for intention, attitude, subjective norm, and PBC, respectively).

#### Personality factors

Participants completed five standard personality scales: Rotter’s Locus of Control Scale [37], the preoccupation and hesitation subscales of the Action Control Scale (ACS) [39], the Generalized Self-Efficacy Scale [40], and the Rosenberg Self-Esteem Scale [36]. These scales generally exhibited satisfactory internal reliability (alphas = .58, .77, .75, .79, and .86, respectively). Although the reliability of the locus of control scale was modest, it was retained because the scale’s reliability and validity are well established [41].

### Analysis strategy

A neural network was used to analyze the data because this strategy has several advantages over traditional regression techniques [42]. Compared to regression, neural networks better handle large numbers of predictors, exceptional data (outliers), and non-linear relationships between predictors and targets. Findings from neural network analyses are also more generalizable to new samples. Neural networks can also identify groups of participants based on the similarity of their predictor-target patterns and therefore indicate what predictors are especially influential for particular types of people (see [42] for demonstrations and discussion). The present study exploited these advantages in order to predict future health behavior from other ongoing lifestyle activities as well as baseline health behaviors, health behavior cognitions, ongoing lifestyle cognitions, and personality factors. Variables were all standardized prior to the analysis. Responses to smoking, getting drunk and eating fast food items were reversed by multiplying by -1 to capture avoidance of health-risk behaviors. Follow-up health behavior was the analysis target and comprised the mean of the relevant z-scores of the five health-related items, providing a single index of *health behavior*. A full account of the neural network analysis is presented in S1 File.

## Results

### Predicting health behavior at follow-up

There were five types of predictors: health behavior cognitions, other lifestyle activities, cognitions about other lifestyle activities, personality factors, and baseline health behaviors. We examined the ability of the neural network to use these variables to predict follow-up health behavior. The predictors were used as inputs to the network, and the follow-up health behavior index was the target that the network was trying to predict. We explored the association between each type of predictor and follow-up health behavior separately across five test waves (one for each predictor type). The predictive utility of all predictor types together was assessed in a sixth test wave, referred to as the *saturated model*.

The network used each predictor type to estimate target health behavior index scores. The association between the network’s estimated and actual health behavior index scores were explored via correlation and regression (Table 2). The regression analyses examined whether other ongoing lifestyle activities and lifestyle cognitions enhanced prediction of health behaviors over and above the effects of health behavior cognitions and personality factors, as well as baseline health behavior. In these analyses, the network’s output (health behavior index estimate) derived from health behavior cognitions (intentions, attitude, subjective norm, and PBC) was entered on the first step. The second step contained the network’s output for personality factors. The third step comprised the network’s output for lifestyle cognitions and activities. Finally, the fourth step contained the network’s output for baseline health behavior.

**Table 2:** Correlations and hierarchical regression analysis of health behavior on predictor types from the neural network.

Step	Predictor	*r*	Adj *R*^*2*^	Adj *R*^*2*^ Change	Step 1 β	Step 2 β	Step 3 β	Step 4 β
1	Health behavior cognitions	.42***	.18	.18***	.42***	.42***	.42***	.13**
2	Personality factors	.09	.18	.00		.07	.04	.04
3	Lifestyle cognitions	.30***					.30***	.16***
	Ongoing lifestyle activities	.47***	.42	.24***			.35***	.19***
4	Baseline health behaviors	.80***	.71	.29***				.65***

Note.

** *p* < .01,

*** *p* < .001.

Correlations showed that health behavior cognitions, lifestyle cognitions, ongoing lifestyle activities, and baseline health behaviors—but not personality factors—were each significantly associated with health behavior scores (Table 2). That is, the respective predictors enabled the network to estimate target health behavior.

In the regression analyses (Table 2), health behavior cognitions explained 18% of the variance on the first step, *F* (1, 209) = 45.59, *p* < .001. At step 2, personality contributed a negligible variance (<1%), *F*_change_ (1, 208) = 1.13, *p = ns*. At step 3, lifestyle cognitions and ongoing activities explained an additional 24% of the variance, *F*_change_ (2, 206) = 44.80, *p* < .001. On the final step, the inclusion of baseline health behaviors accounted for an additional 29% of the variance, *F*_change_ (1, 205) = 205.87, *p* < .001. Importantly, cognitions about, and performance of, other lifestyle activities both significantly predicted health behavior at follow-up even after health behavior cognitions and past behavior had been taken into account (*β*s = .16 and .19, respectively, *p* < .001). The final model accounted for 71% of the variance, *F* (5, 205) = 103.42, *p* < .001.

### Identifying health behavior clusters and their predictors

Data from the saturated model test run, where all sets of predictor variables were presented together to the neural network, were submitted to a two-stage cluster analysis [43, 44]. The aim was to empirically determine groups of participants who were similar in terms of the relationship between predictor variables and health behavior performance at follow-up (see S1 File for technical details). We then determined how each participant group differed from the other groups regarding their health behavior scores and scores on predictor variables. For each variable in each group, we computed Cohen’s *d* [45] for the comparisons between the respective group mean and the mean of the other groups combined. Based on the assumption that effect sizes of medium magnitude or larger would be especially meaningful, a variable was considered salient, i.e., capable of discriminating the relevant health behavior group, if group differences on predictor variables were |*d*| ≥ .50. (A difference of this magnitude is also conventionally significant at *p* < .001.)

The network identified four participant groups according to predictor-output characteristics present in their data (Table 3). As expected, groups differed in their health behavior performance. Group 1 showed high engagement in health behaviors (*d* = 0.98, *n* = 92), group 2 showed moderate engagement (*d* = 0.32, *n* = 50), group 3 showed low engagement (*d* = -1.11, *n* = 64), and group 4 showed very low engagement (*d* = -2.22, *n* = 5). Although this last group was small, this cluster was retained because the pattern of responding was interpretable and provided information about a minority of participants who were committed smokers. Table 3 shows *d*-values exceeding ± 0.5 (Table A in S1 File provides all *d*-values).

**Table 3:** Effect sizes for predictor variables for groups with different levels of engagement in health behaviors.

		Effect Size (*d*)			
		Group 1 (High)	Group 2 (Moderate)	Group 3 (Low)	Group 4 (Very low)
		(n = 92)	(n = 50)	(n = 64)	(n = 5)
**Health behavior cognitions**					
Engage in exercise	Attitude	—	—	—	—
	Subjective norm	0.95	-0.86	—	—
	PBC	0.62	—	—	—
	Intention	0.87	-0.52	-0.54	—
Avoid getting drunk	Attitude	0.59	—	-0.72	—
	Subjective norm	—	—	—	—
	PBC	—	—	-0.55	—
	Intention	0.56	—	-0.61	—
Eat fruit	Attitude	—	—	—	—
	Subjective norm	0.73	-0.77	—	—
	PBC	—	—	—	—
	Intention	0.56	—	-0.67	—
Avoid smoking	Attitude	—	—	—	-2.39
	Subjective norm	0.65	-0.67	—	—
	PBC	—	—	—	-4.20
	Intention	0.53	—	—	-3.18
Avoid fast food	Attitude	—	—	—	—
	Subjective norm	0.54	-0.64	—	-0.52
	PBC	—	—	-0.55	—
	Intention	—	—	—	—
**Personality factors**					
Locus of control		—	—	—	0.50
ACS—preoccupation		—	—	—	0.64
ACS—hesitation		—	—	-0.64	—
Self-esteem		—	—	—	—
Self-efficacy		—	—	—	-0.90
**Lifestyle cognitions**					
Buy a magazine	Attitude	—	—	—	-0.52
	Subjective norm	—	-0.59	—	—
	PBC	—	—	—	—
	Intention	—	—	—	—
Buy a newspaper	Attitude	—	—	—	-0.77
	Subjective norm	0.55	—	—	—
	PBC	—	—	—	—
	Intention	—	—	—	-0.53
Read for pleasure	Attitude	—	—	—	—
	Subjective norm	0.65	-0.92	—	0.53
	PBC	—	—	—	—
	Intention	—	—	—	0.75
Take vitamin pills	Attitude	—	—	—	—
	Subjective norm	—	—	—	—
	PBC	—	—	—	—
	Intention	—	—	—	—
Visit a friend	Attitude	—	—	—	—
	Subjective norm	—	-0.84	—	—
	PBC	—	—	—	—
	Intention	—	-0.57	—	—
Go out for a meal	Attitude	—	—	—	—
	Subjective norm	—	-0.97	—	—
	PBC	—	—	—	—
	Intention	—	-0.56	—	—
Attend all lectures	Attitude	—	—	—	—
	Subjective norm	—	—	—	—
	PBC	—	—	-0.62	-0.59
	Intention	—	—	-0.55	—
7 hours sleep	Attitude	—	—	-0.51	—
	Subjective norm	0.50	—	—	—
	PBC	—	—	—	0.72
	Intention	0.53	—	-0.61	1.03
Buy clothes	Attitude	—	—	—	—
	Subjective norm	0.56	-1.23	—	—
	PBC	—	—	—	—
	Intention	—	—	—	—
Not lying-in past 9.00am	Attitude	—	—	-0.60	—
	Subjective norm	—	—	—	—
	PBC	—	0.56	-0.52	-0.53
	Intention	—	—	-0.62	—
Go to cinema	Attitude	—	—	—	—
	Subjective norm	0.74	-0.83	—	—
	PBC	—	—	—	-0.75
	Intention	0.60	—	—	—
Go for a walk	Attitude	0.66	—	—	—
	Subjective norm	0.89	-1.01	—	—
	PBC	—	—	—	—
	Intention	0.96	-0.61	-0.50	—
Independent study	Attitude	0.68	—	-0.55	—
	Subjective norm	—	—	—	—
	PBC	—	—	—	-0.97
	Intention	0.71	—	-0.61	—
Write a letter	Attitude	—	—	—	0.54
	Subjective norm	—	-0.82	—	—
	PBC	—	—	—	-0.64
	Intention	—	—	—	—
Recycle bottles	Attitude	—	—	—	—
	Subjective norm	0.56	—	—	—
	PBC	—	—	—	—
	Intention	—	—	—	—
Visit the countryside	Attitude	—	—	—	—
	Subjective norm	1.02	-1.33	—	—
	PBC	—	—	—	—
	Intention	0.67	-0.71	—	—
Go to the library	Attitude	—	—	—	-0.51
	Subjective norm	—	-0.64	—	—
	PBC	—	—	—	-0.77
	Intention	—	—	—	—
Avoid eating meat	Attitude	—	—	—	—
	Subjective norm	—	—	—	—
	PBC	—	—	—	—
	Intention	—	—	—	—
Rent a video	Attitude	—	—	—	—
	Subjective norm	—	—	—	—
	PBC	—	—	—	—
	Intention	—	—	—	—
Go shopping	Attitude	—	—	—	—
	Subjective norm	—	-1.02	—	—
	PBC	—	—	—	-0.58
	Intention	—	—	—	-0.62
Visit parents	Attitude	—	—	—	—
	Subjective norm	—	—	—	—
	PBC	—	—	—	-0.67
	Intention	—	—	—	—
Go clubbing	Attitude	—	—	—	—
	Subjective norm	—	-0.64	0.67	—
	PBC	—	—	—	—
	Intention	—	—	0.92	—
Tidy room	Attitude	—	—	—	—
	Subjective norm	—	-0.73	—	—
	PBC	—	—	—	-0.92
	Intention	—	—	—	—
**Ongoing lifestyle activities**					
Buy a magazine		—	—	—	-0.53
Buy a newspaper		—	—	—	-0.77
Read for pleasure		—	—	—	—
Take vitamin pills		—	—	—	0.52
Visit a friend		—	-0.57	0.53	-0.63
Go out for a meal		—	—	—	—
Attend all lectures		—	—	-0.56	-0.62
7 hours sleep		—	—	—	—
Buy clothes		—	—	—	—
Not lying-in past 9.00am		—	—	-0.54	-0.53
Go to cinema		—	—	—	-1.11
Go for a walk		—	—	—	-0.57
Independent study		—	—	—	-0.74
Write a letter		—	—	—	—
Recycle bottles		—	—	—	—
Visit the countryside		—	—	—	—
Go to the library		—	—	—	-1.03
Avoid eating meat		—	—	—	—
Rent a video		—	—	—	—
Go shopping		—	—	—	-0.72
Visit parents		—	—	—	—
Go clubbing		—	—	0.73	—
Tidy room		—	—	—	—
**Baseline health behaviors**					
Engage in exercise		0.90	—	-0.72	—
Avoid getting drunk		0.52	0.64	-0.98	-0.51
Eat fruit		0.77	—	-1.03	—
Avoid smoking		—	—	—	-4.07
Avoid eating fast food		—	—	-0.85	—

*Note*. Only values of *d* exceeding ± 0.5 are shown.

Group 1 = high engagement in health behavior group, Group 2 = moderate engagement group, Group 3 = low engagement group, Group 4 = very low engagement group,

PBC = perceived behavioral control, ACS = Action Control Scale [39].

As predicted, different variables were salient for different groups (see Table 3). For participants with high levels of engagement in health behaviors at follow-up (group 1), health cognitions were salient: Intentions and subjective norms were strong for four out of the five health behaviors. Despite their frequent performance of health behaviors, there was no difference in how often group 1 participants engaged in the 23 ongoing lifestyle activities compared to the other groups; *d* values for these activities did not exceed ± 0.5. This is consistent with the idea that these potentially competing activities were managed efficiently. Interestingly, participants in this group also felt normative pressure to engage in those other activities.

A different pattern emerged for participants with moderate engagement in health behaviors (group 2). The factor that most clearly discriminated this group was low levels of normative influence. Weak subjective norms were apparent for four of the five health behaviors and for 13 out of the 23 other ongoing activities (57%). Like group 1, ongoing lifestyle activities and personality variables were not salient for this group.

Consistent with predictions, ongoing lifestyle activities were salient for the low engagement in health behavior group (group 3). In particular, participants in this cluster showed a high degree of social engagement (visiting a friend, going clubbing); they were also likely to engage in behaviors that were antithetical to their academic goals (lying in past 9.00 a.m., not attending all lectures). A number of cognitions about the health behaviors were salient, notably weak motivation as indicated by low intention for three of the five behaviors. Intention was also salient for 6 of the 23 other activities (26%), five of which indexed low motivation, especially for behaviors related to studying. One personality variable distinguished this group from the other participants, namely, low scores on the hesitation sub-scale of the ACS. This finding indicates that participants in group 3 experienced problems in initiating action.

Group 4 comprised participants with very low levels of health behavior at follow-up. The group’s small size (*n* = 5) necessitates caution when interpreting the findings, though we note a number of observations. The baseline health behavior and allied cognitions data indicated that these participants were committed smokers. We expected that other ongoing activities would be especially salient for this group. This proved to be the case, but the effect was in the opposite direction to what was anticipated. Group 4 engaged in *fewer*, not more, alternative activities. Participants were less likely to read (magazines, newspapers), socialize (visit friends, go for walks, go to the cinema), and pursue their academic goals (attend lectures, avoid lying in, independent study) compared to the other groups. The only lifestyle behavior that they engaged in more frequently than other participants was taking vitamin supplements. Lifestyle cognitions were also salient, and there was evidence of low perceived control for 9 of the 23 behaviors (39%). Personality factors were salient for this group. Participants exhibited low self-regulatory capability as shown by elevated external locus of control [37], low generalized self-efficacy [40], and high scores on the ACS-preoccupation subscale [35]. High preoccupation scores indicate a tendency to ruminate about setbacks and failure rather than get started on one’s goal pursuit [39]. Thus, the personality data and the findings for cognitions about other ongoing behaviors indicated this small cluster of participants exhibited poor action control.

## Discussion

This study aimed to understand health behaviors in context, and in particular, how people coordinate health actions in relation to other ongoing activities. Cavallo and Fitzsimons [46] pointed out that “most investigations into multiple goals have usually examined only two goals” (p. 293). To better capture the reality of striving for multiple goals, the present research assessed five health behaviors together with 23 other ongoing behaviors that participants might undertake during the same follow-up period. Two key novel findings emerged. First, other ongoing lifestyle activities influenced health behavior performance. Cognitions about, and enactment of, other ongoing activities significantly predicted health behavior at follow-up. These lifestyle variables accounted for a significant increment in the variance explained in behavior, over and above the influence of past behavior and cognitions about the health behaviors. Second, there were notable individual differences in the extent to which other ongoing activities and the other predictor variables influenced health behaviors. For participants who exhibited moderate and high levels of health behavior at follow-up, the behavior-specific cognitions and past performance were salient. On the other hand, cognitions about, and the enactment of other activities were salient for participants who exhibited low and very low engagement in health behaviors. Moreover, both low engagement groups were characterized by poor action control as indexed by the ACS [39]. These findings suggest that ongoing activities are not mere extraneous variables that health behavior theories can afford to ignore. Lifestyle cognitions and behaviors forge a consequential context for health behavior performance—a milieu that matters, and matters differently for different people.

The present study has theoretical, practical, and analytic implications for health behavior research. The theoretical implications are four-fold. First, our findings support other evidence [47] questioning the “sufficiency assumption” of the theory of planned behavior (TPB)–the idea that extraneous variables only influence the target behavior via their effects on attitude, subjective norm, perceived behavioral control, or intention [3]. It should be noted that the present study supported the predictive validity of TPB variables. The cognitions specified by the TPB explained 18% of the variance in health behaviors. This finding is in line with McEachan et al.’s [6] meta-analysis of the TPB and health behaviors, and suggests that our use of neural network analysis did not disadvantage the TPB’s power to predict behavior. Nonetheless, cognitions about, and performance of, other ongoing activities directly predicted behavior even after TPB variables and prior behavior had been taken into account, and explained a meaningful increment in the variance. These findings suggest that the TPB may need to be extended to take account of the important influence of other ongoing activities on health behaviors.

Second, the present study tested, and found support for, a prediction derived from counteractive control theory [31] that other ongoing lifestyle activities would be especially influential among participants who do not engage in health behaviors. Fishbach et al. [31] used priming paradigms to demonstrate that, for people who are successful at self-regulation, the presence of competing goal pursuits (temptations) was automatically countered by increased activation of the health goal—unlike people who are unsuccessful at self-regulation. In the present study, cluster analysis identified groups of participants who were more versus less successful at performing health behaviors. We observed equivalent effects to those obtained by Fishbach et al. [31]. In particular, even though participants who successfully engaged in health behaviors (group 1) were no less likely to engage in other lifestyle behaviors compared to the other groups, these participants still held strong health-related intentions. Conversely, among participants who did not engage health behaviors (group 3), cognitions and performance in relation to other ongoing behaviors were highly salient and health-related intentions and PBC were weaker. This is consistent with the idea that these participants have less efficient mechanisms for handling competing goals. Thus, the present findings would seem to support counteractive control theory in a field setting.

The third theoretical implication concerns the important role of poor self-regulatory capacity among participants who did not engage in health behaviors. Participants with low levels of engagement in health behaviors (group 3) were characterized by difficulties in initiating action as indicated by low scores on the hesitation sub-scale of the ACS. Although the number of participants with very low levels of engagement in health behaviors was small (group 4), findings indicated problems in action initiation with an external locus of control, low levels of generalized self-efficacy, and rumination about setbacks and failure (high scores on the preoccupation sub-scale of the ACS). The influence of these personality variables was observed only when distinct groups were analyzed. We observed no significant overall association between personality variables and health behavior performance in the regression analyses. Findings may suggest that the influence of personality factors, and self-regulatory capability in particular, is subtle and may be masked in analyses of whole-sample data. Self-regulatory capability is important but only for participants who need to change their health behaviors—the key target group for interventions (see also [32]).

The final theoretical implication concerns the influence of subjective norms among participants who showed moderate and high levels of engagement in health behaviors. This finding was not anticipated, not least because subjective norm typically is a weaker predictor of intentions and behavior compared to attitude and PBC (e.g., [6]). Subjective norms were salient for participants showing moderate and high levels of engagement in health behaviors, and were as salient as intention for high-engagement participants. The salience of subjective norm also differed for the moderate versus high engagement groups. Whereas the *d*-value for subjective norms was positive for 4 out of the 5 health behaviors among participants with high levels of engagement, the *d*-value was *negative* for 4 out of the 5 health behaviors among participants with moderate levels of engagement. An equivalent pattern was observed for subjective norm in relation to other ongoing activities. These findings may suggest that social pressure to engage in health behaviors is important and is especially important for the transition from moderate to high levels of engagement in health behaviors. One possible reason why the important influence of subjective norm was more apparent here compared to previous research is that neural networks can better handle non-linear relationships compared to traditional regression analysis. Future research might therefore do well to test non-linear as well as linear relationships when testing the role of subjective norms.

The present findings also have implications for practice, and in particular for interventions to promote health behavior change. A useful innovation of the present research was to identify different clusters indicative of health behavior engagement and discover the key factors that characterize those clusters. Our findings suggest that different intervention strategies may be effective according to participants’ current health behavior performance. Targeting subjective norms could be effective among participants who currently exhibit moderate levels of performance (see, e.g., [48, 49]). For participants with low levels of engagement in health behaviors, interventions might be better targeted at weak intentions and low PBC regarding health behaviors. This group also had positive cognitions about, and high levels of engagement in, socializing (e.g., visiting friends, clubbing) and negative cognitions about, and low levels of engagement in, academic work (e.g., studying, attending lectures). This suggests that interventions may need to address participants’ broader life goals and seek to alter the priority that is attached to socializing versus academic and health behaviors. Participants with low levels of engagement in health behaviors also exhibited problems initiating action. As implementation intentions are known to facilitate getting started on one’s goal pursuits (review by [50]), this self-regulation tool could prove beneficial for low engagement participants [51–53].

The present research also has analytic implications as it suggests that neural networks are a valuable technique that could be exploited more fully in health behavior research (e.g. [42, 54]). Neural network analysis has advantages compared to traditional regression models in that it can handle large numbers of predictors, outliers, and non-linear relationships. Moreover, cluster analysis can be applied to data from the network in order to empirically identify subgroups of interest; these subgroups can then be compared in terms of their predictor-outcome relations. Thus, neural network analysis could prove a valuable strategy for identifying *what* variables are important *for whom* and so inform the design of future behavior change interventions [55].

### Limitations

As with any new program of research, the present study has limitations that should be acknowledged. First, participants were university students and so caution should be exercised in extrapolating the findings to the other populations; further tests are needed using more representative and clinical samples to warrant generalizations. Second, behavior was followed up after two weeks. This short time-frame is not a problem for the current study because the essential question was not about *habit* change per se, but rather variations in health behavior resulting from wider contextual factors within a given time frame. However, future research may want to explore effects of context and habit change with an extended follow-up period. Third, we acknowledge that questionnaire responses may be subject to some recall bias and error when participants considered their behavior and cognitions over the preceding period. Fourth, to reduce participant burden, behavior was assessed by single-item self-report measures. Future studies would do better to deploy objective measures of health behaviors (e.g., cotinine tests to measure smoking, pedometers to measure exercise). Fifth, we were able to assess only a limited set of behaviors and personality variables in the present study. Improved indices of dietary behavior (e.g., changes in BMI), measures of other important health behaviors (e.g., sleep, condom use), and laboratory tests of self-control and executive function would be valuable supplements in future research. Furthermore, broader contextual factors, not considered here, such as SES, ethnicity, and place (e.g., urban vs. rural) will affect opportunities, time and physical resources needed for behavioral engagement. Future research may want to explore how such structural factors may moderate, or be mediated by, the influence of ongoing lifestyle activities on health behaviors. Finally, the sample size was modest. Nonetheless, the network was able to extract meaningful information, and the early stopping procedure (described in S1 File) gives us confidence that findings can be applied more widely. However, caution is especially warranted in drawing strong conclusions about participants with very low engagement in health behaviors. This group contained a very small number of participants, and additional studies are needed to corroborate the present findings.

### Conclusion

Notwithstanding these limitations, the present research offers new insights into how milieu matters for health behaviors. Health actions typically are undertaken in the same settings and during the same time periods as other ongoing lifestyle activities. Moreover, health behaviors and other lifestyle activities both draw upon the same limited pool of self-control resources for their successful execution (e.g., [56]). The present findings suggest that health behavior theories will be enriched and the prediction of health behaviors will be improved when other lifestyle activities are considered alongside the focal health behavior(s). Most important, taking account of the many, varied lifestyle intentions and behaviors that people regularly manage affords new targets for health behavior change interventions, especially for people who most need to change their behavior.

## Supporting information

S1 File Supporting information for milieu matters. (DOCX)
