# Supplementary material for: Milieu matters: Evidence that ongoing lifestyle activities influence health behaviors
Source: PLoS One. 2017 Jun 29;12(6):e0179699. doi: 10.1371/journal.pone.0179699 (PMC5491030; doi:10.1371/journal.pone.0179699)
Supplement: S1 File — (DOCX) [file pone.0179699.s001.docx]

**Supporting Information for**

**Milieu Matters: Evidence that Ongoing Lifestyle Activities Influence Health Behaviors**

Rob Lowe^1^, Paul Norman^2^, Paschal Sheeran^3*^

^1^ Swansea University, Swansea, UK

^2^ University of Sheffield, Sheffield, UK

^3^ University of North Carolina-Chapel Hill, Chapel Hill, North Carolina, USA

*Corresponding author

Paschal Sheeran, Department of Psychology, University of North Carolina-Chapel Hill, Chapel Hill, Davie Hall, NC 27599, USA, email: psheeran@unc.edu

The study used a neural network to analyze associations between predictor and outcome variables. What follows is an overview of neural networks, a description of the set-up of the network used in the analyses, and an outline of the approach taken to explore the network’s outputs.

**Neural Networks**

Neural networks grew from attempts to understand information processing in biological systems [[1](#_ENREF_1),[2](#_ENREF_2)] and are modeled in contemporary research using computer simulations [[3-5](#_ENREF_3)]. In psychology, neural networks are useful tools for understanding cognitive processes because they exhibit characteristics similar to human information processing (e.g., noise tolerance, self-organization, parallel processing). A neural network of the sort used in the paper is essentially a learning machine that finds regularities in relationships between input (predictor) variables and a specified target variable. The network identifies an underlying function that best describes the input-target relationship (analogous to fitting a line in regression).

In addition to modeling cognitive processes, neural networks can be used as statistical tools for analyzing complex real-world data (e.g., [[6-8](#_ENREF_6)]). Neural networks have a number of advantages over conventional regression in terms of handling non-linearity, outliers (exceptions), and large numbers of predictors. Furthermore, neural networks are able to identify prototype groups within samples, and this was exploited in the current research. Each prototype group would be expected to differ from the others in the sample according to relationships between predictor and target variables; that is, certain variables will be more or less important to some groups than to other groups. For a discussion and examples of the advantages of neural networks for analyzing complex real-world data, see [[8](#_ENREF_8)].

The current study used a neural network to determine the extent to which an index of follow-up health behaviors could be predicted by other ongoing lifestyle activities and baseline measures of health behavior, personality, health behavior cognitions, and cognitions about other lifestyle activities.

**Network Setup**

The study used a feed-forward neural network trained through error backpropagation. It was modeled in version 3.1 of the PDP++ neural network simulation software [[9](#_ENREF_9),[10](#_ENREF_10)]. Such networks usually contain three layers: an input layer where activation enters the system, a hidden layer which contains the network’s internal representation of the data, and an output layer where the network produces a response. Layers comprise one or more units (or nodes) capable of rudimentary computations. Units in each layer are linked to those in adjacent layers via adjustable (weighted) connections that govern the spread of activation through the system. Connections are where the effects of learning input-output associations are stored.

Figure S1 shows the basic structure of the network used in the current study. The ovals represent layers, each of which comprised a number of individual units (not shown). The input layer contained five sub-layers, each receiving one of the input types: health behavior cognitions, personality factors, other lifestyle cognitions, other lifestyle activities, baseline health behaviors. Each unit in each sub-layer represented a specific individual predictor variable. Hence, the number of input units varied between sub-layers because the number of constituents for each class of variable differed (e.g., there were 5 personality factors and 20 health behavior cognitions with 5 and 20 units in their respective input sub-layers). Units in each input sub-layer were fully connected to units in a corresponding hidden layer.

Output Layer

(1 unit)

Hidden Layers

(3/6 units)

Input Layers

Personality OL Cognitions Health H Beh. Other

(5 units) (92 units) Behavs. Cognitions Activities

(5 units) (20 units) (23 units)

**S1 Fig. Structure of the Neural Network**

*Note* OL Cognitions = other lifestyle cognitions; Health Behavs. = baseline health behaviors;

H Beh. Cognitions = health behavior cognitions.

The hidden layer was sub-divided according to input type (personality factors, other lifestyle cognitions, and so on) because we wanted to retain differentiation of these psychological constructs within the network’s internal representation. This enabled us to determine, during the testing phase (see below), the ability of each construct to independently predict the output. Four hidden sub-layers were size 3 units, and one sub-layer (relating to other lifestyle cognitions) was size 6 units because of the greater number of inputs this sub-layer received (see section on constraining the network below). Units in each of the five hidden sub-layers were fully connected to the output layer. The output layer comprised a single unit where the network produced a response - in this case, its reconstruction (estimate) of the follow-up health-behavior index score.

Units in the hidden sub-layers were set to have an activity limit between 0 and 1, whereas the output unit had the limit set to ± 2.5 (to accommodate the full range of the health-behavior target data). Connection weights had no upper or lower bounds. Before training, weights were given a random value (*M* = 0, variance = 0.5). The *learning rate* was set to 0.001. This determined how fast weights were updated; a low value was used to ensure that learning remained stable. Simple *weight decay* was set to 0.0001; this helped the network ignore spurious inputs that did not meaningfully contribute to learning [[11](#_ENREF_11)]. *Momentum* was set to 0.2; this helped the network avoid a less-than-optimum solution and counter undue oscillations in weight change during learning [[12](#_ENREF_12)].

Prior to entry into the neural network, all predictor variables (health behavior cognitions, personality factors, other lifestyle cognitions, other lifestyle activities, and baseline health behaviors) were standardized. The follow-up measure (the output target) was a single index of the five health protective behaviors and comprised the mean of the respective behavior z-scores.

**Network Training**

To identify patterns in the data set, the network was first *trained*. Here, it learned (via weight change) to assign a particular output when presented with a particular input. The evolved weight matrix retained information that best distinguished patterns in the data while eliminating noise.

Training involved presenting the data to the network multiple times. During each presentation, the predictor variables for each participant were entered into the input layer and the network was tasked with using this information to predict that person’s target variable (health behavior index). Error between the network’s estimate and the actual target was used for adjusting connection weights. This aimed to reduce error in future presentations. Thus, across multiple presentations, the network’s estimate converged towards the desired target. Each presentation of the whole data set is known as an *epoch*. For the current study, network training comprised 200 epochs (see Constraining the Network below).

**Network Testing**

Following training, the network was tested on what it had learned. No weight change occurred during testing. The network was presented with each participant’s input data, and an estimate of their target output (follow-up health behavior index score) was produced. Assuming the network had derived useful information during training then the output should be relatively accurate. Test activity on hidden and output units was recorded for subsequent analysis.

The study explored the extent to which each input type (health behavior cognitions, personality factors, other behavior cognitions, and so on) could predict follow-up health behavior. To derive the network’s estimates of follow-up behavior based on each of these input types, there were five test waves – one for each input type. This involved presenting participant data for each input type – separately – to the network, and recording activity on the corresponding hidden and output layers. Finally, the predictive utility of all inputs collectively was assessed; here data for all input variables were presented concurrently in a sixth test wave (the *saturated model*).

**Unpacking the Network’s Output**

It was important to understand the network’s behavior in terms of a) the degree to which it was able to predict follow-up health behavior based on the predictors (its *accuracy*) and b) *how* it was able to make its predictions.

To determine the network’s *accuracy* in predicting health behavior, we compared the network’s estimate of health behavior (derived from the specific types of predictor) with target health behavior index scores. As described in the main article, this was done via regression analysis. Here, the independent variables comprised the network’s estimates of health behavior arising from the specified predictor(s), and the dependent variable comprised the target health behavior index score. If the network was able to make good estimates based on specific types of predictor, then the regression coefficient between the network’s estimated and actual follow-up health behavior should be high. Table 1 in the main article summarizes the regression results for the separate predictors.

Next, we needed to determine *how* the network made its predictions. In essence, the network identified participant groups based on commonalities in relationships between their predictor variables and their engagement in follow-up health behavior. The relevant procedure is described below.

**Identifying Participant Groups and Key Predictors**

Groups of people may be similar in terms of their lifestyle and motivations, and the effect that these factors have on their health behaviors. Statistically, such groups would comprise people with similar predictor-outcome relationships. In terms of the neural network, this would be reflected by groups of people whose data produced similar activation patterns on the hidden and output layers. The following analysis aimed to identify these groups, and to identify which specific input variables were most or least important for each group. Specifically, activation on hidden and output units was subjected to cluster analysis to identify prototype groups. Following this, mean scores for study variables were compared across prototype groups to determine those particularly strong (or weak) for each group.

We analyzed data from the saturated model test run (where all inputs were presented together). Hidden and output layer activations for each participant were recorded. These were converted to *z*-scores and the entire set submitted to a two-stage cluster analysis [[13](#_ENREF_13),[14](#_ENREF_14)] involving a Ward followed by a K-means method. We used the Ward analysis (squared Euclidian distance) to identify the number of clusters (from the agglomeration schedule and dendrogram) as well as the cluster centroids. This information was then used for the subsequent K-Means analysis, which required the number of clusters to be specified, and in which the cluster centroids were used as non-random starting seeds (see [[13](#_ENREF_13)]). Cluster membership (i.e., assignment to participant groups) was taken from the K-Means analysis.

Having clustered participants according to the similarity of their hidden and output unit activations, further analyses determined how each participant group differed from other groups in terms of both their output (health behavior index scores) and input (scores on predictor variables). This was done by comparing each variable in each group with the mean for that variable combined across the remaining groups. For each comparison, we computed Cohen’s *d* [[15](#_ENREF_15),[16](#_ENREF_16)], which are displayed in Table S1. A difference was considered meaningful if an effect size was of medium magnitude or larger. That is to say, a variable was considered salient if comparison differences yielded *d* ≥ +/-.50 (these values are significant at *p <* .001 for *N* = 211).

**S1 Table. Effect sizes (*d*) for Predictor Variables for Groups with Different Levels of Engagement in Health Behaviors**

|  | | **Effect Size (*d*)** | | | |
| --- | --- | --- | --- | --- | --- |
|  |  | **gp1** | **gp2** | **gp3** | **gp4** |
|  |  | **(n=92)** | **(n=50)** | **(n=64)** | **(n=5)** |
| **Health behavior cognitions** | | | | | |
| Engage in exercise | Att | 0.34 | 0.01 | -0.37 | -0.20 |
|  | SN | 0.95 | -0.86 | -0.31 | -0.31 |
|  | PBC | 0.62 | -0.40 | -0.32 | -0.13 |
|  | Intention | 0.87 | -0.52 | -0.54 | 0.02 |
| Avoid getting drunk | Att | 0.59 | 0.13 | -0.72 | -0.41 |
|  | SN | 0.37 | -0.12 | -0.28 | -0.36 |
|  | PBC | 0.27 | 0.42 | -0.55 | -0.20 |
|  | Intention | 0.56 | 0.01 | -0.61 | -0.44 |
| Eat fruit | Att | 0.27 | -0.05 | -0.29 | 0.19 |
|  | SN | 0.73 | -0.77 | -0.07 | -0.14 |
|  | PBC | 0.01 | 0.39 | -0.34 | 0.33 |
|  | Intention | 0.56 | 0.07 | -0.67 | -0.08 |
| Avoid smoking | Att | 0.39 | 0.28 | -0.46 | -2.39 |
|  | SN | 0.65 | -0.67 | -0.19 | 0.22 |
|  | PBC | 0.27 | 0.45 | -0.28 | -4.20 |
|  | Intention | 0.53 | 0.08 | -0.36 | -3.18 |
| Avoid eating fast food | Att | 0.30 | 0.11 | -0.45 | -0.02 |
|  | SN | 0.54 | -0.64 | 0.00 | -0.52 |
|  | PBC | 0.25 | 0.40 | -0.55 | -0.25 |
|  | Intention | 0.13 | 0.36 | -0.49 | 0.15 |
| **Personality factors** | | | | | |
| Locus of control | | -0.18 | 0.05 | 0.12 | 0.50 |
| ACS - preoccupation | | 0.19 | -0.04 | -0.25 | 0.64 |
| ACS - hesitation | | 0.39 | 0.22 | -0.64 | -0.33 |
| Self-esteem | | 0.06 | -0.02 | -0.02 | -0.24 |
| Self-efficacy | | 0.39 | -0.24 | -0.16 | -0.90 |
| **Lifestyle cognitions** | | | | | |
| Buy a magazine | Att | 0.07 | -0.21 | 0.16 | -0.52 |
|  | SN | 0.23 | -0.59 | 0.16 | 0.44 |
|  | PBC | -0.41 | 0.42 | 0.16 | 0.23 |
|  | Intention | -0.16 | 0.00 | 0.19 | -0.03 |
| Buy a newspaper | Att | 0.38 | -0.12 | -0.27 | -0.77 |
|  | SN | 0.55 | -0.42 | -0.23 | -0.33 |
|  | PBC | -0.17 | 0.38 | -0.06 | -0.12 |
|  | Intention | 0.23 | 0.14 | -0.34 | -0.53 |
| Read for pleasure | Att | 0.16 | 0.16 | -0.32 | 0.18 |
|  | SN | 0.65 | -0.92 | -0.02 | 0.53 |
|  | PBC | 0.22 | -0.07 | -0.23 | 0.36 |
|  | Intention | 0.28 | -0.12 | -0.28 | 0.75 |
| Take vitamin pills | Att | 0.14 | 0.00 | -0.18 | 0.09 |
|  | SN | 0.12 | -0.37 | 0.16 | 0.37 |
|  | PBC | -0.06 | 0.16 | -0.07 | 0.14 |
|  | Intention | 0.03 | -0.12 | 0.03 | 0.37 |
| Visit a friend | Att | 0.04 | 0.01 | -0.07 | 0.16 |
|  | SN | 0.37 | -0.84 | 0.36 | -0.17 |
|  | PBC | 0.05 | -0.44 | 0.38 | 0.07 |
|  | Intention | 0.28 | -0.57 | 0.15 | 0.12 |
| Go out for a meal | Att | -0.05 | 0.14 | -0.05 | 0.07 |
|  | SN | 0.34 | -0.97 | 0.39 | 0.32 |
|  | PBC | 0.19 | -0.22 | 0.04 | -0.43 |
|  | Intention | 0.27 | -0.56 | 0.16 | 0.04 |
| Attend all lectures | Att | 0.44 | -0.03 | -0.44 | -0.19 |
|  | SN | 0.32 | -0.23 | -0.19 | 0.44 |
|  | PBC | 0.44 | 0.31 | -0.62 | -0.59 |
|  | Intention | 0.50 | 0.01 | -0.55 | -0.02 |
| 7 hours sleep | Att | 0.24 | 0.29 | -0.51 | 0.41 |
|  | SN | 0.50 | -0.19 | -0.37 | -0.35 |
|  | PBC | 0.24 | -0.01 | -0.32 | 0.72 |
|  | Intention | 0.53 | -0.05 | -0.61 | 1.03 |
| Buy clothes | Att | -0.02 | -0.31 | 0.28 | 0.19 |
|  | SN | 0.56 | -1.23 | 0.27 | 0.37 |
|  | PBC | -0.11 | -0.03 | 0.17 | -0.02 |
|  | Intention | -0.01 | -0.25 | 0.24 | -0.10 |
| Not lying-in past 9.00am | Att | 0.42 | 0.06 | -0.60 | 0.22 |
|  | SN | 0.44 | -0.42 | -0.15 | 0.19 |
|  | PBC | 0.13 | 0.56 | -0.52 | -0.53 |
|  | Intention | 0.31 | 0.27 | -0.62 | 0.14 |
| Go to cinema | Att | 0.23 | 0.12 | -0.31 | -0.33 |
|  | SN | 0.74 | -0.83 | -0.10 | -0.28 |
|  | PBC | 0.04 | 0.15 | -0.05 | -0.75 |
|  | Intention | 0.60 | -0.37 | -0.31 | -0.46 |
| Go for a walk | Att | 0.66 | -0.27 | -0.47 | -0.16 |
|  | SN | 0.89 | -1.01 | -0.12 | 0.00 |
|  | PBC | 0.40 | -0.18 | -0.29 | 0.08 |
|  | Intention | 0.96 | -0.61 | -0.50 | -0.05 |
| Independent study | Att | 0.68 | -0.16 | -0.55 | -0.45 |
|  | SN | 0.33 | -0.34 | -0.02 | -0.41 |
|  | PBC | 0.41 | 0.14 | -0.47 | -0.97 |
|  | Intention | 0.71 | -0.15 | -0.61 | -0.32 |
| Write 2 letters | Att | 0.30 | -0.11 | -0.30 | 0.54 |
|  | SN | 0.48 | -0.82 | 0.16 | 0.09 |
|  | PBC | 0.01 | 0.18 | -0.09 | -0.64 |
|  | Intention | 0.36 | -0.41 | -0.05 | -0.09 |
| Recycle bottles | Att | 0.24 | 0.01 | -0.27 | -0.10 |
|  | SN | 0.56 | -0.49 | -0.18 | -0.15 |
|  | PBC | 0.13 | -0.23 | 0.09 | -0.42 |
|  | Intention | 0.26 | -0.30 | -0.06 | 0.09 |
| Visit the countryside | Att | 0.47 | -0.30 | -0.25 | -0.11 |
|  | SN | 1.02 | -1.33 | -0.05 | -0.08 |
|  | PBC | 0.17 | -0.29 | 0.01 | 0.42 |
|  | Intention | 0.67 | -0.71 | -0.19 | 0.26 |
| Go to the library | Att | 0.30 | 0.16 | -0.43 | -0.51 |
|  | SN | 0.39 | -0.64 | 0.13 | 0.07 |
|  | PBC | 0.06 | 0.01 | 0.02 | -0.77 |
|  | Intention | 0.22 | -0.25 | 0.00 | -0.32 |
| Avoid eating meat | Att | 0.33 | -0.21 | -0.22 | 0.09 |
|  | SN | 0.36 | -0.50 | 0.00 | -0.18 |
|  | PBC | 0.19 | 0.10 | -0.24 | -0.44 |
|  | Intention | 0.34 | -0.23 | -0.24 | 0.24 |
| Rent a video | Att | 0.04 | 0.10 | -0.08 | -0.31 |
|  | SN | 0.48 | -0.50 | -0.13 | -0.17 |
|  | PBC | -0.07 | -0.09 | 0.20 | -0.37 |
|  | Intention | 0.22 | -0.37 | 0.10 | -0.28 |
| Go shopping | Att | 0.09 | -0.14 | 0.03 | -0.06 |
|  | SN | 0.40 | -1.02 | 0.42 | -0.33 |
|  | PBC | -0.06 | -0.14 | 0.30 | -0.58 |
|  | Intention | 0.03 | -0.34 | 0.36 | -0.62 |
| Visit parents | Att | 0.38 | -0.07 | -0.40 | 0.31 |
|  | SN | 0.41 | -0.27 | -0.23 | -0.09 |
|  | PBC | 0.02 | 0.19 | -0.10 | -0.67 |
|  | Intention | 0.13 | 0.14 | -0.30 | 0.16 |
| Go clubbing | Att | -0.14 | -0.30 | 0.44 | 0.42 |
|  | SN | -0.07 | -0.64 | 0.67 | 0.04 |
|  | PBC | -0.26 | -0.07 | 0.49 | -0.43 |
|  | Intention | -0.43 | -0.33 | 0.92 | -0.27 |
| Tidied room | Att | 0.19 | -0.16 | -0.03 | -0.35 |
|  | SN | 0.40 | -0.73 | 0.23 | -0.40 |
|  | PBC | 0.13 | -0.02 | 0.06 | -0.92 |
|  | Intention | 0.35 | -0.46 | 0.01 | 0.04 |
| **Ongoing lifestyle activities** | | | | | |
| Buy a magazine | | -0.05 | 0.13 | -0.02 | -0.53 |
| Buy a newspaper | | 0.04 | 0.27 | -0.23 | -0.77 |
| Read for pleasure | | 0.29 | 0.04 | -0.45 | 0.29 |
| Take vitamin pills | | 0.02 | -0.12 | 0.01 | 0.52 |
| Visit a friend | | -0.06 | -0.57 | 0.53 | -0.63 |
| Go out for a meal | | -0.18 | -0.07 | 0.30 | -0.39 |
| Attend all lectures | | 0.49 | 0.21 | -0.56 | -0.62 |
| 7 hours sleep | | 0.14 | 0.23 | -0.38 | 0.14 |
| Buy clothes | | -0.19 | 0.12 | 0.13 | -0.14 |
| Not lying-in past 9.00am | | 0.32 | 0.32 | -0.54 | -0.53 |
| Go to cinema | | 0.28 | -0.11 | -0.16 | -1.11 |
| Go for a walk | | 0.37 | -0.13 | -0.32 | -0.57 |
| Independent study | | 0.42 | -0.15 | -0.29 | -0.74 |
| Write 2 letters | | 0.25 | -0.02 | -0.30 | 0.12 |
| Recycle bottles | | 0.18 | -0.12 | -0.13 | -0.23 |
| Visit the countryside | | 0.41 | -0.22 | -0.31 | -0.25 |
| Go to the library | | 0.28 | 0.04 | -0.29 | -1.03 |
| Avoid eating meat | | 0.30 | -0.27 | -0.17 | 0.29 |
| Rent a video | | 0.12 | -0.49 | 0.19 | 0.03 |
| Go shopping | | -0.14 | -0.21 | 0.40 | -0.72 |
| Visit parents | | 0.24 | -0.22 | -0.24 | 0.47 |
| Go clubbing | | -0.46 | -0.34 | 0.73 | 0.25 |
| Tidied room | | 0.27 | -0.13 | -0.20 | -0.18 |
| **Baseline health behaviors** | | | | | |
| Engage in exercise | | 0.90 | -0.46 | -0.72 | -0.04 |
| Avoid getting drunk | | 0.52 | 0.64 | -0.98 | -0.51 |
| Eat fruit | | 0.77 | 0.15 | -1.03 | -0.42 |
| Avoid smoking | | 0.30 | 0.35 | 0.12 | -4.07 |
| Avoid eating fast food | | 0.46 | 0.42 | -0.85 | -0.27 |
| **Follow-up health behaviors** | | | | | |
| Engage in exercise | | 0.63 | -0.10 | -0.65 | -0.43 |
| Avoid getting drunk | | 0.50 | 0.49 | -0.89 | -0.35 |
| Eat fruit | | 0.58 | 0.08 | -0.69 | -0.59 |
| Avoid smoking | | 0.20 | 0.32 | 0.17 | -2.66 |
| Avoid eating fast food | | 0.54 | 0.10 | -0.66 | -0.10 |

*Note*. gp1 = high engagement in health behavior group, gp2 = moderate engagement group, gp3 = low engagement group, gp4 = very low engagement group, Att = Attitude, SN = subjective norm, PBC = perceived behavioral control, Int = intention, ACS = Action Control Scale [[17](#_ENREF_17)].

**Constraining the Network**

We wanted to ensure the network did not over-fit the data. Over-fitting occurs when the system extracts individual input-output relationships but fails to identify the principles underlying these relationships. Three approaches were adopted to minimize the risk of over-fitting. First, the network employed weight decay (described above) so as to better ignore spurious inputs. Second, the size (in terms of number of units) of each hidden sub-layer was kept small. This constrained the computing power of the network. Pre-testing indicated that the sizes chosen could accommodate the inputs without a notable decline in predictive ability. This meant that the network had sufficient capacity to compute the data, without being over-powered. Third, the number of training epochs was constrained. Training occurred across 200 epochs; this number being determined by preliminary analysis using an *early stopping* procedure (e.g., [[18](#_ENREF_18)]).

The early stopping procedure is a technique for identifying the point at which over-fitting begins, and thus identifies when to stop training. It is a split-half method where a portion of the data is used for training, and what is learned is applied to predicting the critical dependent variable in the remaining data portion. In the current study, data from two thirds of participants was used for training (n = 141), the remainder for testing (n = 70). Group assignment was random. The network was trained across 300 epochs in batches of 20 epochs. After each 20-epoch batch, the test data was applied to the network. Each test run involved presenting the predictor variables for the test sample and computing the output-target error. This determined whether information extracted (i.e., weight change) from the training sample to that point could be applied to those in the test sample.

After each complete train-test run (300 epochs), weights were randomized, and the whole procedure repeated. The average error across 20 complete runs was plotted; the error curve dropped across initial training batches but then bottomed-out and began to rise slightly. The point of rise was where over-fitting began (i.e., where information from the training sample could no longer be generalized to the test sample). The lowest error point was at 200 training epochs which was the number of epochs selected for network training in the main study run.

**References**

1. Hebb DO (1949) The organization of behavior. New York: Wiley.

2. Mcculloch WS, Pitts W (1990) A Logical Calculus of the Ideas Immanent in Nervous Activity (Reprinted from Bulletin of Mathematical Biophysics, Vol 5, Pg 115-133, 1943). Bulletin of Mathematical Biology 52: 99-115.

3. Ellis R, Humphreys G (1999) Connectionist psychology: A text with readings. Hove, East Sussex, UK: Psychology Press.

4. McClelland JL, Rumelhart DE, the PDP Research Group (1986) Parallel distributed processing: Explorations in the microstructure of cognition, volume 2: Psychological and biological models. London: MIT Press.

5. Rumelhart DE, McClelland JL, the PDP Research Group (1986) Parallel distributed processing: Explorations in the microstructure of cognition, volume 1: Foundations. London: MIT Press.

6. DeTienne KB, DeTienne DH, Joshi SA (2003) Neural networks as statistical tools for business researchers. Organizational Research Methods 6: 236-265.

7. Lowe R, Bennett P, Walker I, Milne S, Bozionelos G (2003) A connectionist implementation of the theory of planned behavior: Association of beliefs with exercise intention. Health Psychology 22: 464-470.

8. Walker I, Milne S (2005) Exploring function estimators as an alternative to regression in psychology. Behav Res Methods 37: 23-36.

9. O'Reilly RC, Dawson CK, McClelland JL, Carnegie Mellon University (1995-2003) The PDP++ software [Computer software].

10. Dawson CK, O'Reilly RC, McClelland JL, Carnegie Mellon University (1995-2003) The PDP++ software users manual [Computer software manual].

11. O’Reilly RC, Munakata Y (2000) Computational explorations in cognitive neuroscience: Understanding the mind by simulating the brain. London: MIT Press.

12. Callan R (1999) The essence of neural networks. Harlow, UK: Pearson.

13. Clatworthy J, Hankins M, Buick D, Weinman J, Horne R (2007) Cluster analysis in illness perception research: A Monte Carlo study to identify the most appropriate method. Psychology & Health 22: 123-142.

14. Milligan GW (1980) An examination of the effect of six types of error perturbation on fifteen clustering algorithms. Psychometrika 45: 325-342.

15. Cohen J (1988) Statistical power analysis for the behavioral sciences (2nd ed.). Hillsdale, NJ: Erlbaum.

16. Cohen J (1992) A Power Primer. Psychological Bulletin 112: 155-159.

17. Kuhl J (1994) Action versus state orientation: Psychometric properties of the Action Control Scale (ACS-90). In: Kuhl J, Beckmann J, editors. Volition and personality: Action versus state orientation. Seattle, WA: Hogrefe & Huber. pp. 47-59.

18. Finnoff W, Hergert F, Zimmermann HG (1993) Improving Model Selection by Nonconvergent Methods. Neural Networks 6: 771-783.
